# First Clinical Results of the Merit WRAPSODY™ Cell-Impermeable Endoprosthesis for Treatment of Access Circuit Stenosis in Haemodialysis Patients

**DOI:** 10.1007/s00270-021-03001-1

**Published:** 2021-11-09

**Authors:** James Gilbert, Jason Rai, David Kingsmore, John Skousen, Nikolaos Ptohis

**Affiliations:** 1 Oxford Transplant Centre, Oxford University Hospitals NHS Foundation Trust, Old Road, Headington, OX3 7LE Oxford UK; 2 Queen Elizabeth University Hospital, Glasgow, UK; 3 Merit Medical Systems, Inc, South Jordan, Utah USA; 4 General Hospital of Athens “G.Gennimatas”, Athens, Greece

We were delighted to read the letter from P Bream, J Beecham-Chick and M Lessne in response to publication of our manuscript entitled ‘First Clinical Results of the Merit WRAPSODY™ Cell-Impermeable Endoprosthesis for Treatment of Access Circuit Stenosis in Haemodialysis Patients’ [1]. The respondents have suggested that there has been improper use of the endoprosthesis and make this comment based on the clinical example shown in Fig. 3 of the manuscript. They rightly highlight that stent placment has extended through the cephalic arch and into the mid subclavian vein but suggest that this could have been a complication rather than recognise how well placed the stent is to treat the stenosis and ensure in line flow. The respondents also go on to suggest that this case represents a poor example of vein preservation for the lifetime of the patient due to the potential ‘jailing’ of the basilic and axillary veins.

Whilst they are completely right to raise such concerns, I would like to outline the important complex clinical decision-making processes that we as clinicians went through and, more importantly, the backstory to the patient we have treated in this case as that may help the respondent’s and wider readers to realise that this was far from ‘a poor example of vein preservation’ but in fact a great example of considering the K-DOQI Kidney life plan recommendations [2].

The images in Fig. 3 were of a frail 77-year-old individual who had been on dialysis for 6 years and had no transplant option. This patient had a background of hypertensive nephropathy, severe COPD from lifelong smoking and limited exercise tolerance. There had been two previous access creation attempts before a left brachiocephalic AVF was established in 2015. This developed Cephalic Arch Stenosis in 2018 and was treated with Balloon Angioplasty on two occasions. In our institution, there is a weekly Multi-Disciplinary Team (MDT) meeting at which the access surgeon, nephrology team and radiologist discuss all images in the context of the patient to make an appropriate plan. The MDT felt that stenting for this case was appropriate, and the least invasive option given the severe frailties and limited prognosis. The WRAPSODY stent was placed in Sept 2019 and provided a patent circuit and ongoing good dialysis adequacy throughout the study period. This patient recently died from an exacerbation of COPD. It is our view that this was a case of an excellent MDT working together to decide on the ‘right access option at the right time in the right patient for all the right reasons’ [2].

As an MDT, we always take into consideration future AV access options for our patients and do recognise that this is critically important especially if any treatment or intervention of an existing circuit lesion could possibly prevent future new circuit creation. Such decision-making will, however, be done in the context of the patient and all possible renal replacement therapy options including transplantation. In some circumstance, preserving future options will not be ‘right’ or in the best interest of a specific patient’s ESKD life plan as this case highlighted.
